# 668 Influence of Infrastructure on Global Burn Outcomes: Analysis of the WHO Global Burn Registry

**DOI:** 10.1093/jbcr/iraf019.297

**Published:** 2025-04-01

**Authors:** Daniel Najafali, Megan Najafali, Hilary Liu, José Arellano, Saeid Rezaei, Logan Galbraith, Christopher Fedor, Mare Kaulakis, Quincy Tran, Francesco Egro

**Affiliations:** Carle Illinois College of Medicine; Loyola University Chicago Stritch School of Medicine; University of Pittsburgh Medical Center; University of Pittsburgh Medical Center; University of Pittsburgh Medical Center; Northeast Ohio Medical University; University of Pittsburgh School of Medicine; University of Pittsburgh School of Medicine; University of Maryland School of Medicine; University of Pittsburgh Medical Center

## Abstract

**Introduction:**

Burn injury management varies in areas with limited infrastructure, where resources for supporting care are often inconsistent. Understanding the limitations and barriers to burn care delivery is crucial to improving health outcomes in developing regions. We hypothesize that burn patients in areas with less robust healthcare infrastructure have worse outcomes compared to those in settings with better infrastructure.

**Methods:**

The entirety of the WHO Global Burn Registry was leveraged for this analysis to examine infrastructure and resources. Descriptive statistics captured facility-level information relating to burn admissions, computerization, critical care availability, inpatient bed capacity, internet connectivity, nutritional support capabilities, operating theatre availability, facility ownership, physiotherapy and rehabilitation services, population served, specialist capabilities, specialized treatment units, urban or rural location, and wound care capabilities.

**Results:**

The majority of burn cases were managed at institutions with moderate admissions (N=4,919, 53%) defined as a maximum of 160 annual burn cases. Access to computerization was available at 71% of institutions, while those with limited critical care capacity managed 1,341 (15%) cases. Most cases (93%) had reliable access to surgical infrastructure for burn patients. The majority also had reliable access to physical therapy (87%). In low-resource regions, access to operating rooms, rehabilitation services, specialist care, and advanced plastic and reconstructive surgery was significantly lower (P< 0.001). Mortality rates and discharge with impairment were significantly higher in settings lacking these resources (P< 0.001).

**Conclusions:**

Burn patients treated in facilities with limited access to critical care, specialized surgical capabilities, and rehabilitation services in low-resource regions experienced worse outcomes.

**Applicability of Research to Practice:**

Improving resource allocation and developing infrastructure in low-resource settings are critical for reducing disparities in burn outcomes. Efforts could include training programs to build local capacity for burn surgery and advocating for international collaboration. Investing in infrastructure improvements could reduce the global burden of burn injuries and significantly enhance long-term patient recovery, particularly in low-resource regions.

**Funding for the Study:**

N/A

